# Supplementary material for: Andrographolide and Its 14-Aryloxy Analogues Inhibit Zika and Dengue Virus Infection
Source: Molecules. 2020 Oct 30;25(21):5037. doi: 10.3390/molecules25215037 (PMC7662321; doi:10.3390/molecules25215037)

Andrographolide and Its 14-Aryloxy Analogues Inhibit Zika and Dengue Virus Infection

Feng Li[1,†], Wipaporn Khanom[2,†], Xia Sun[1], Atchara Paemanee[3], Sittiruk Roytrakul[3], Decai Wang[1], Duncan R. Smith[2,*], Guo-Chun Zhou[1,*]

[1] School of Pharmaceutical Sciences, Nanjing Tech University, Nanjing 211816, Jiangsu, China

[2] Molecular Pathology Laboratory, Institute of Molecular Biosciences, Mahidol University, 25/25 Phuttamonthon Sai 4, Salaya, Nakorn Pathom 73170, Thailand

[3] Proteomics Research Laboratory, Genome Technology Research Unit, National Center for Genetic Engineering and Biotechnology, National Science and Technology Development Agency, 113 Thailand Science Park, Phahonyothin Road, Khlong Nueng, Khlong Luang, Pathumthani 12120, Thailand

†These authors contributed equally to the work.

*Correspondence should be addressed to gczhou@njtech.edu.cn (GCZ) or duncan_r_smith@hotmail.com (DRS).

Contents

Figures S1-S19 --- pp 2-20
Chemistry general information --- pp21
NMR and HPLC spectra ---pp 22-27

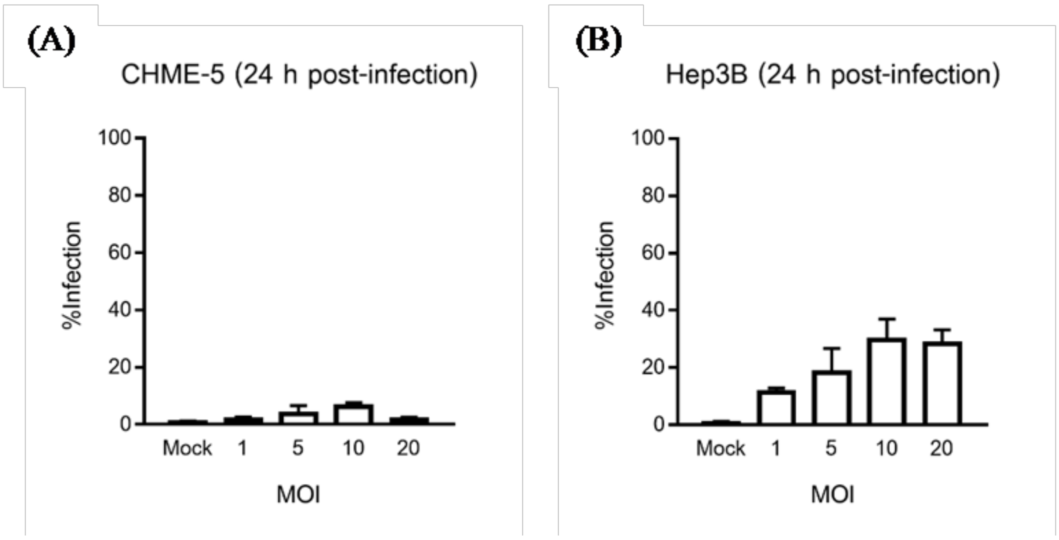

Figure S1. Percentage of infection of ZIKV-T and DENV-2 on different cell lines. CHME-5 (A) and Hep3B (B) cells were infected with ZIKV-T at MOI 1, 5, 10, and 20 for 24 h. The cells were harvested to determine the level of infection by flow cytometry. All experiments were undertaken independently in triplicate.

Figure S2. Cytotoxicity of ZAD-1 (A), ZAD-2 (B), ZAD-3 (C), and andrographolide (D) to BHK-21 (baby hamster kidney) cells. BHK-21 cells were incubated with various concentrations of each compound for 24 hrs followed by MTT cell viability assay. The experiment was undertaken independently in 4 replicates. DMSO at the same concentrations was used as a control. 10% ethanol, PBS, and milli-Q water were used as a positive control of cell death. The bars show mean ± SD.

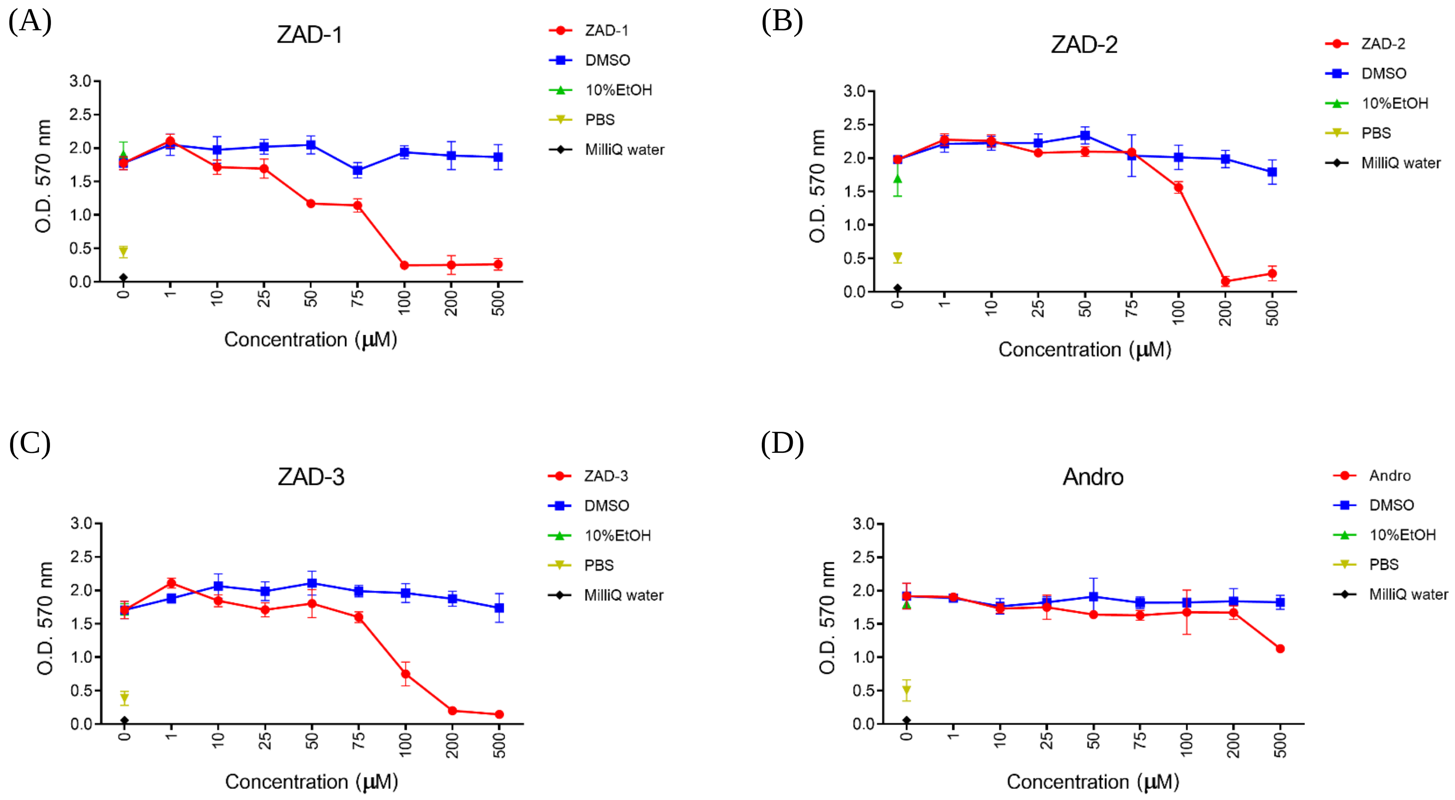

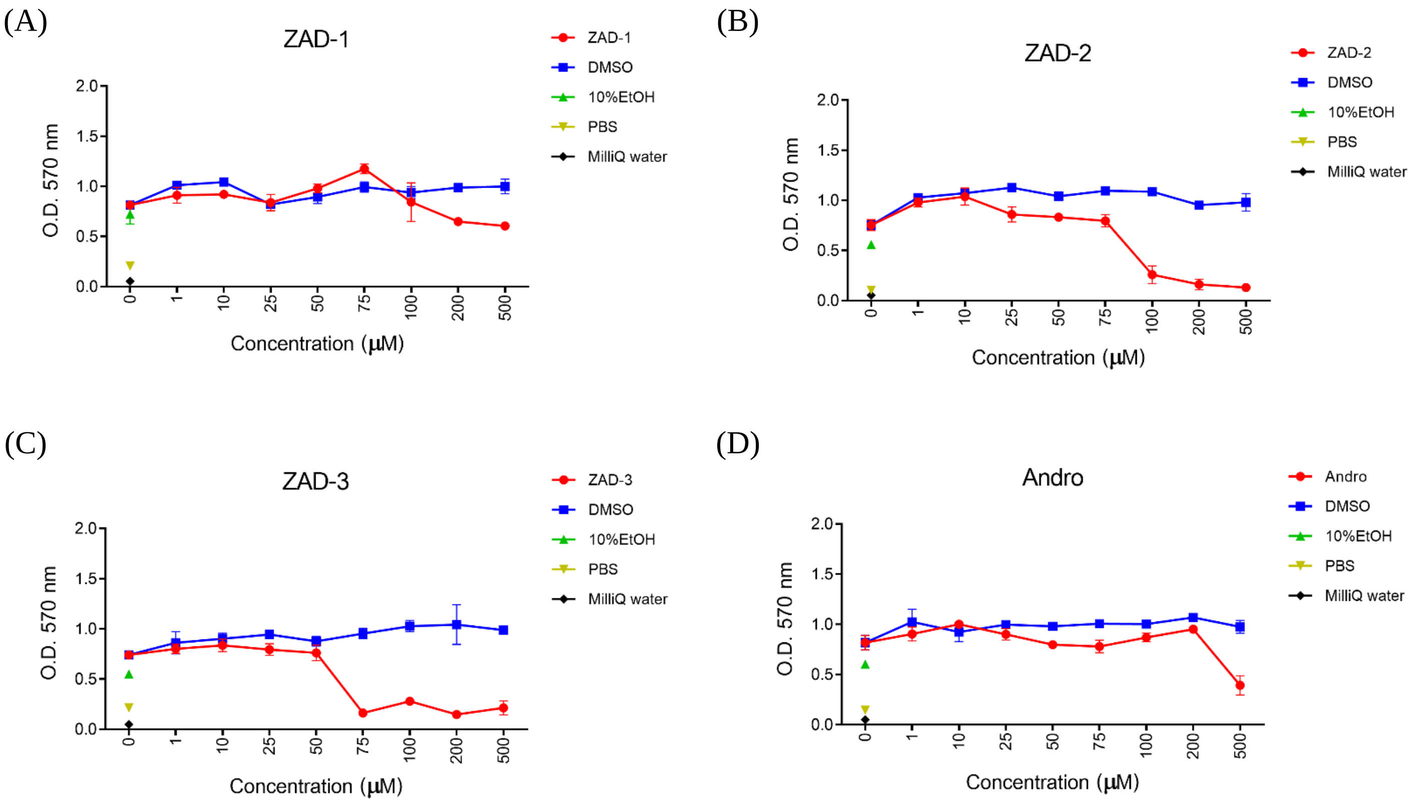

Figure S3. Cytotoxicity of ZAD-1 (A), ZAD-2 (B), ZAD-3 (C), and andrographolide (D) to Vero (monkey kidney) cells. Vero cells were incubated with various concentrations of each compound for 24 hrs followed by MTT cell viability assay. The experiment was undertaken independently in 4 replicates. DMSO at the same concentrations was used as a control. 10% ethanol, PBS, and milli-Q water were used as a positive control of cell death. The bars show mean ± SD.

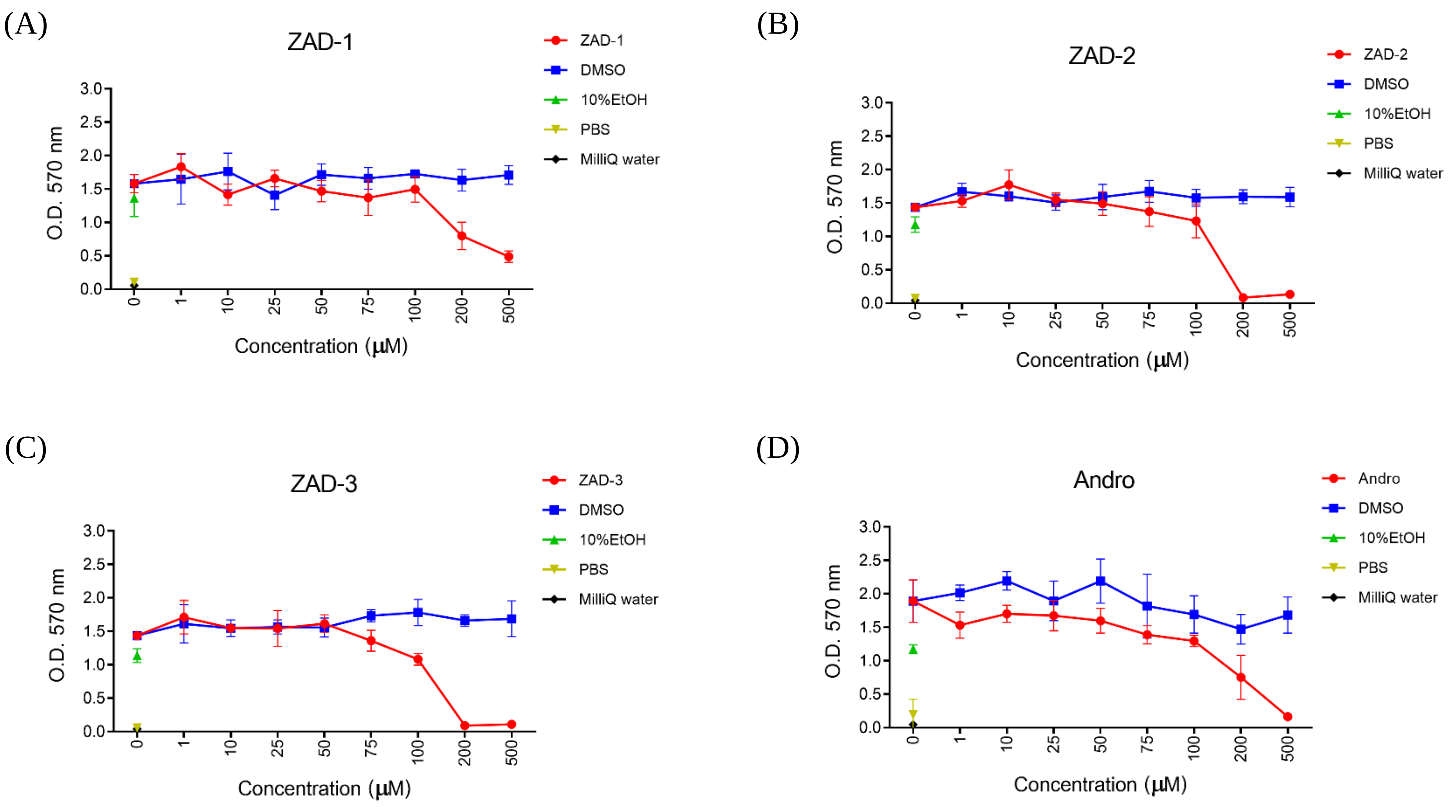

Figure S4. Cytotoxicity of ZAD-1 (A), ZAD-2 (B), ZAD-3 (C), and andrographolide (D) to A549 (human lung carcinoma) cells. A549 cells were incubated with various concentrations of each compound for 24 hrs followed by MTT cell viability assay. The experiment was undertaken independently in 4 replicates. DMSO at the same concentrations was used as a control. 10% ethanol, PBS, and milli-Q water were used as a positive control of cell death. The bars show mean ± SD.

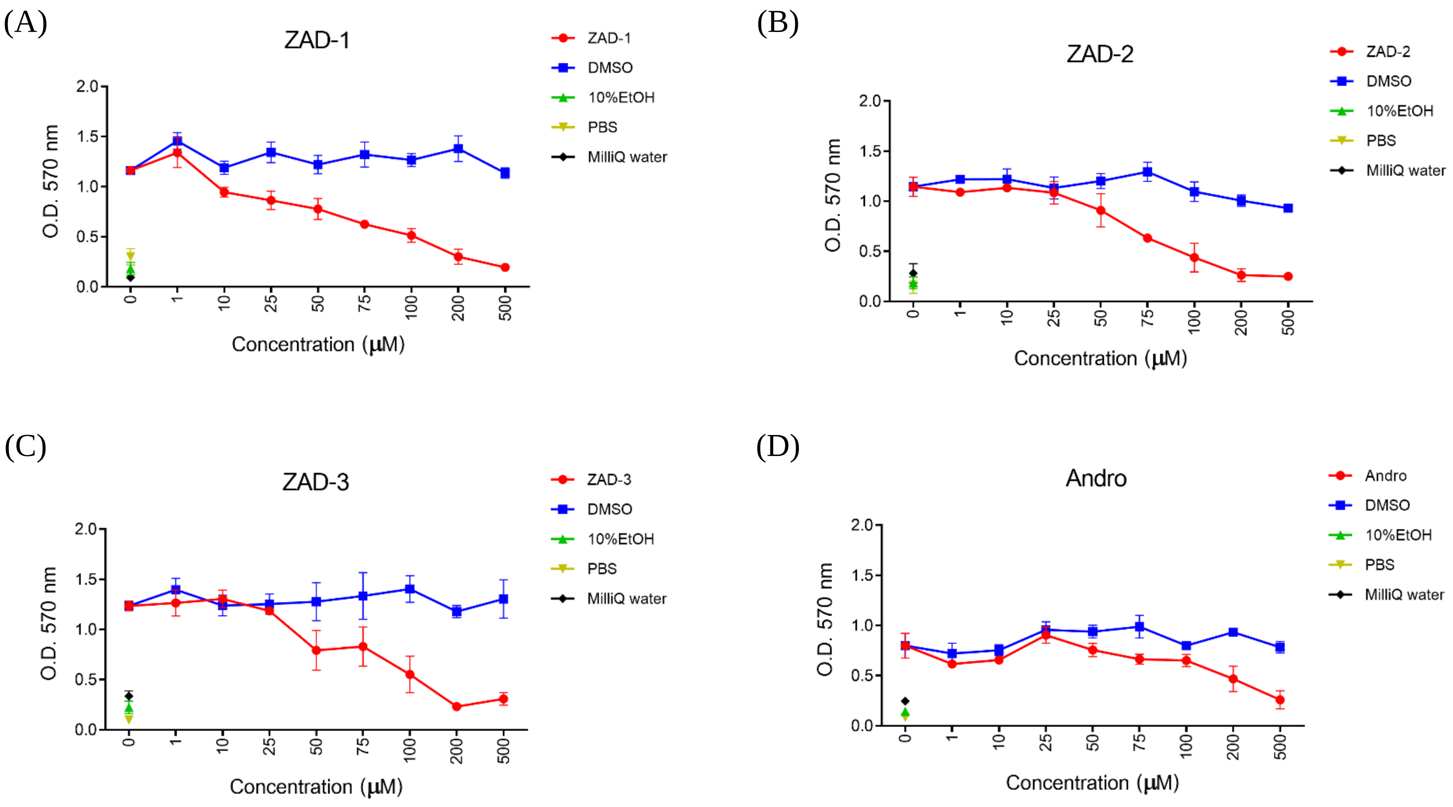

Figure S5. Cytotoxicity of ZAD-1 (A), ZAD-2 (B), ZAD-3 (C), and andrographolide (D) to HEK293T/17 (human embryonic kidney) cells. HEK293T/17 cells were incubated with various concentrations of each compound for 24 hrs followed by MTT cell viability assay. The experiment was undertaken independently in 4 replicates. DMSO at the same concentrations was used as a control. 10% ethanol, PBS, and milli-Q water were used as a positive control of cell death. The bars show mean ± SD.

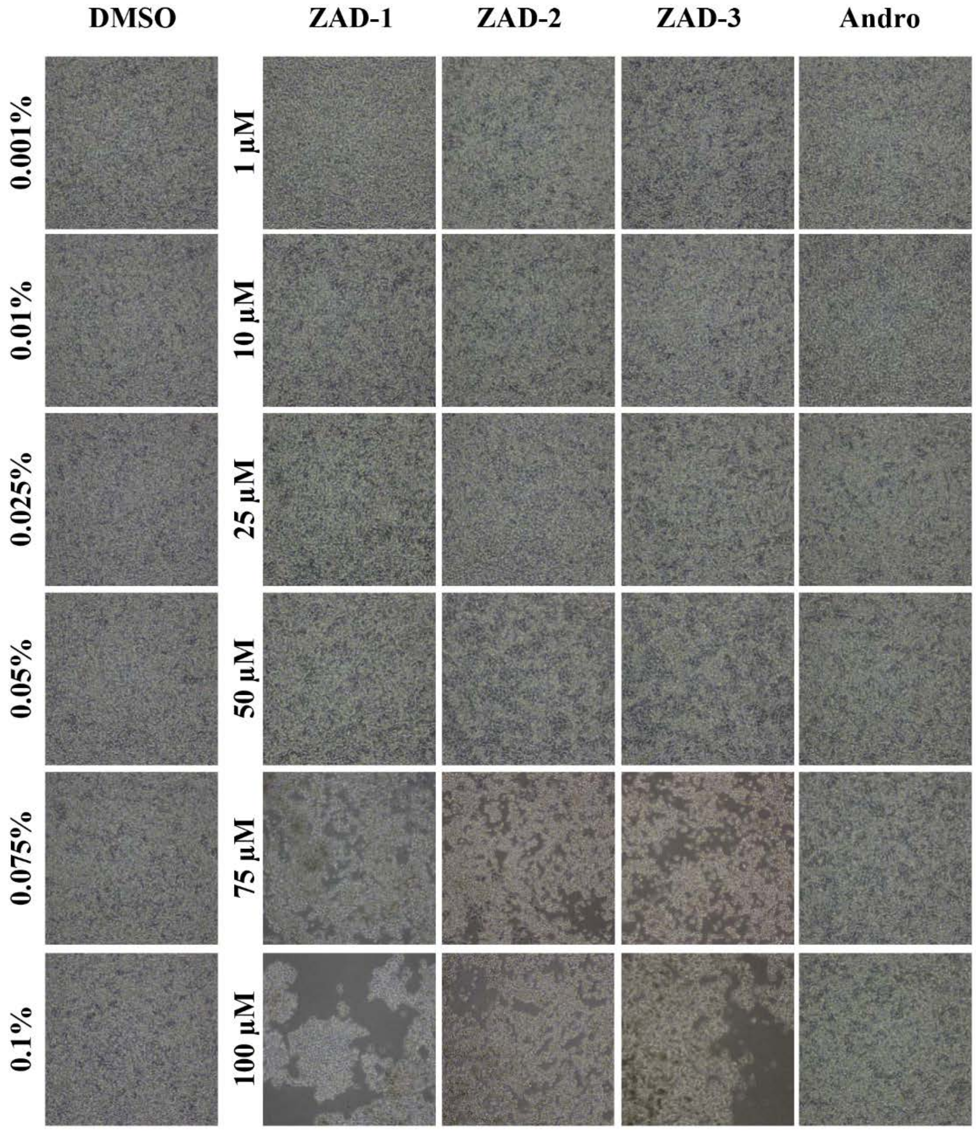

Figure S6. The morphological changes affected by ZAD-1, ZAD-2, ZAD-3 and andrographolide on BHK-21 cells observed under a light microscope. BHK-21 cells were incubated with various concentrations of andrographolide or andrographolide analogues in parallel with DMSO as a control for 24 hrs then the morphological changes were observed under a light microscope.

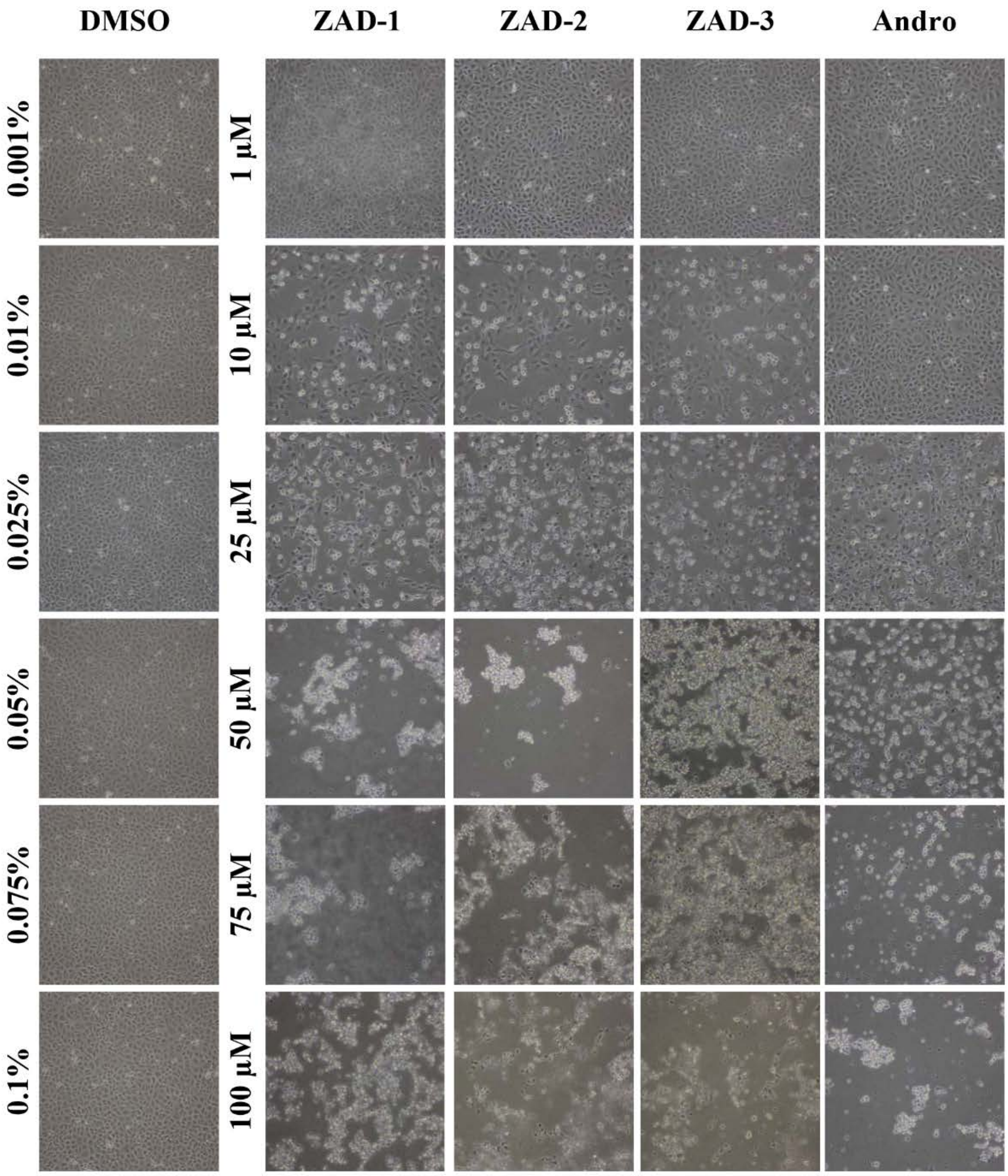

Figure S7. The morphological changes affected by ZAD-1, ZAD-2, ZAD-3 and andrographolide on Vero cells observed under a light microscope. Vero cells were incubated with various concentrations of andrographolide or andrographolide analogues in parallel with DMSO as a control for 24 hrs then the morphological changes were observed under a light microscope.

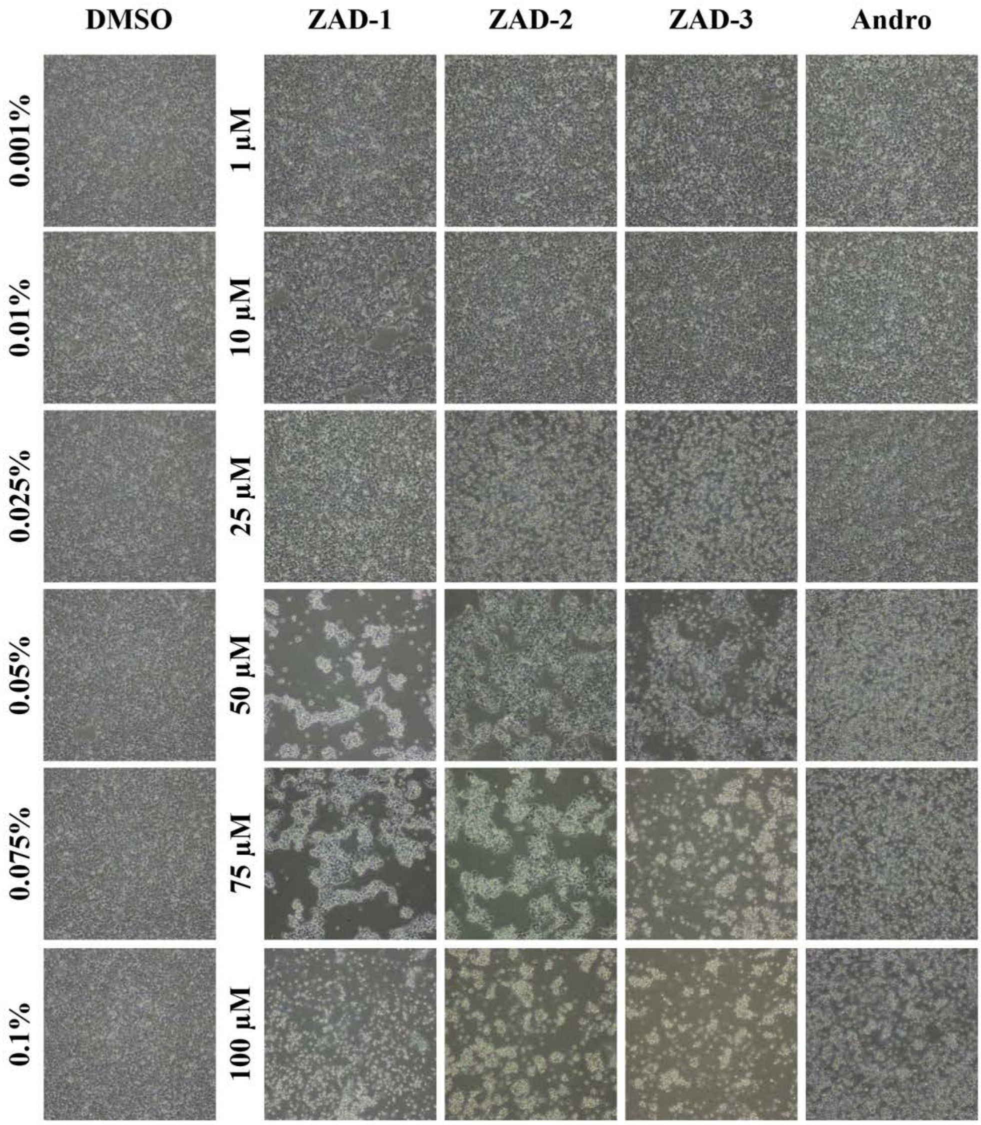

Figure S8. The morphological changes affected by ZAD-1, ZAD-2, ZAD-3 and andrographolide on HEK293T/17 cells observed under a light microscope. HEK293T/17 cells were incubated with various concentrations of andrographolide or andrographolide analogues in parallel with DMSO as a control for 24 hrs then the morphological changes were observed under a light microscope.

(A) (B) (C)

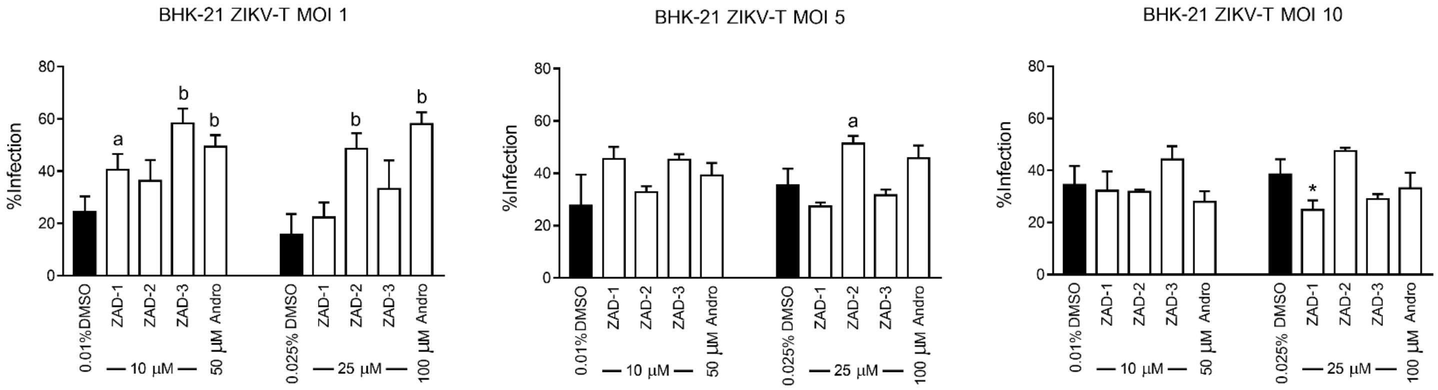

Figure S9. Effect of andrographolide and its analogues (ZAD-1, ZAD-2 and ZAD-3) on ZIKV infected BHK21 cells. The cells were infected with ZIKV at MOI of 1 (A), 5 (B) and 10 (C). After removing unbound virus, cells were treated with drugs at various concentrations. At 24 hrs post-treatment, cells were harvested and analyzed the level of infection by flow cytometry. The experiment was undertaken independently in triplicate. The error bars show mean ± SD (* and a indicates *p* value < 0.05, b indicates p value <0.01)

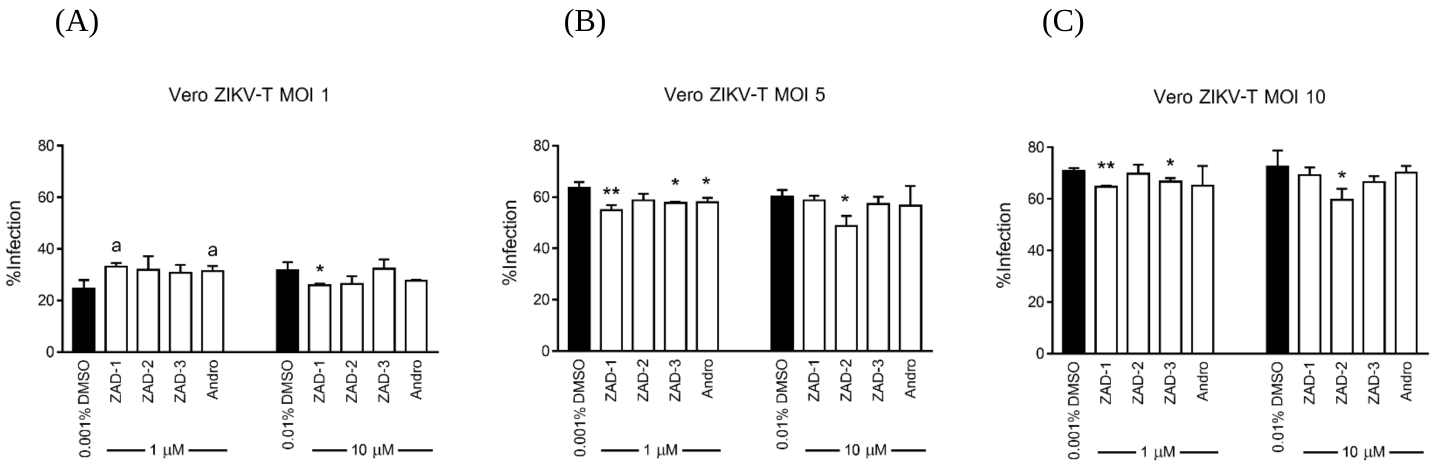

Figure S10. Effect of andrographolide and its analogues (ZAD-1, ZAD-2 and ZAD-3) on ZIKV infected Vero cells. The cells were infected with ZIKV at MOI of 1 (A), 5 (B) and 10 (C). After removing unbound virus, cells were treated with drugs at various concentrations. At 24 hrs post-treatment, cells were harvested and analyzed the level of infection by flow cytometry. The experiment was undertaken independently in triplicate. The error bars show mean ± SD (* and a indicates *p* value < 0.05; ** indicates p value <0.01)

(A) (B) (C)

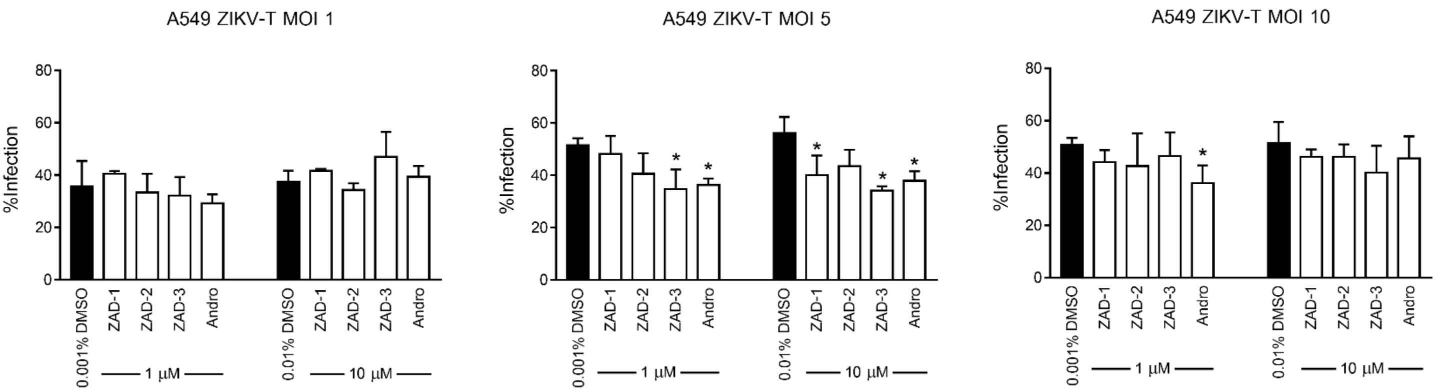

Figure S11. Effect of andrographolide and its analogues (ZAD-1, ZAD-2 and ZAD-3) on ZIKV infected A549 cells. The cells were infected with ZIKV at MOI of 1 (A), 5 (B) and 10 (C). After removing unbound virus, cells were treated with drugs at various concentrations. At 24 hrs post-treatment, cells were harvested and analyzed the level of infection by flow cytometry. The experiment was undertaken independently in triplicate. The error bars show mean ± SD (* means *p* value < 0.05)

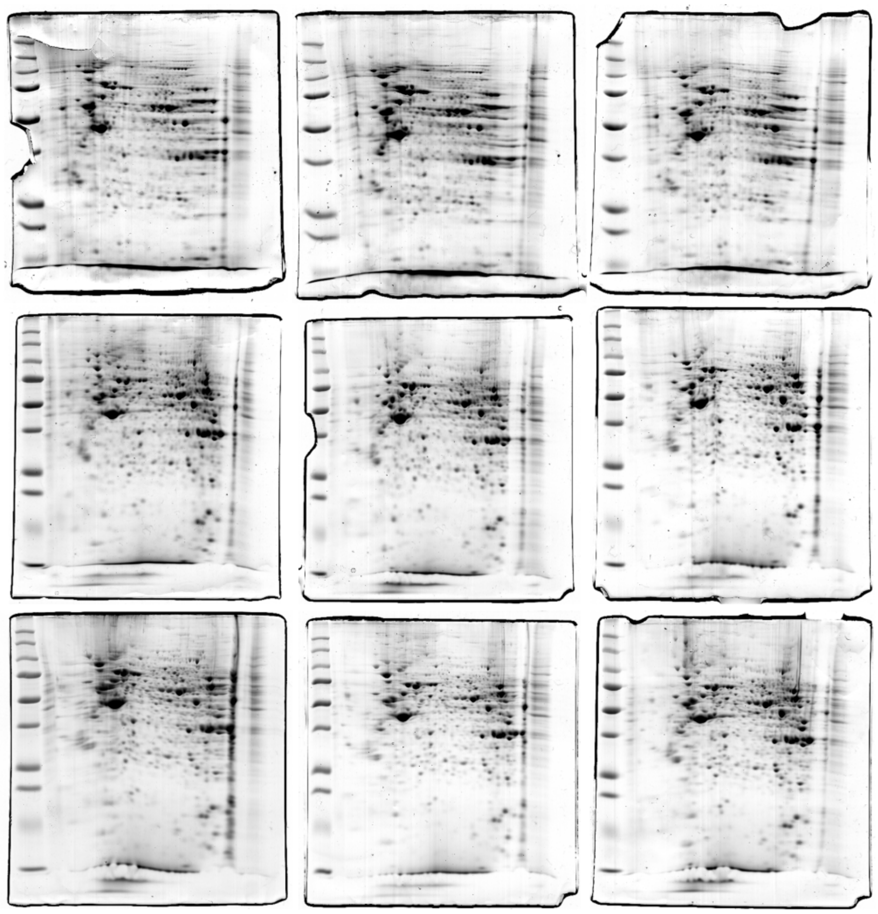

Figure S12. Replicate 2D-gels

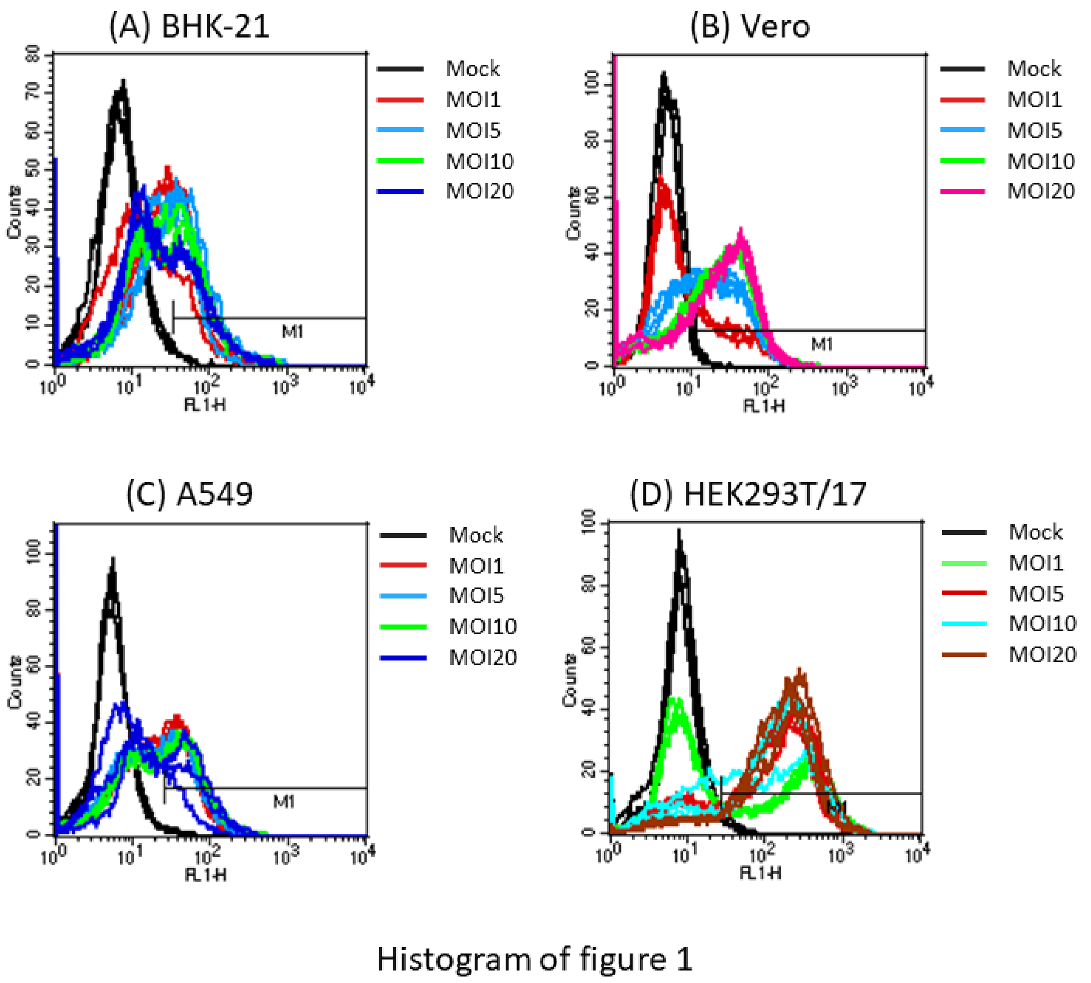

Figure S13. Raw flow cytometry data from Figure 1.

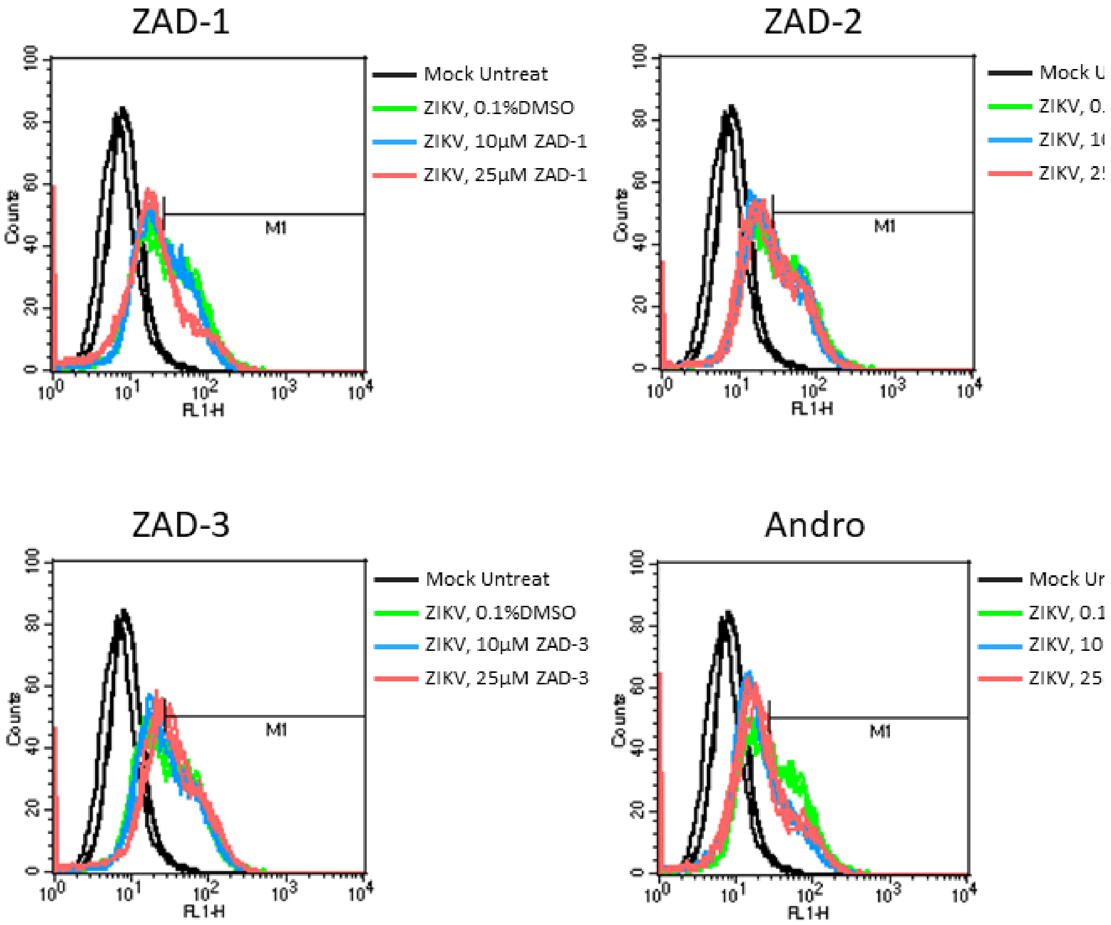

Histogram of figure 3(A)

Figure S14. Raw flow cytometry data from Figure 3A.

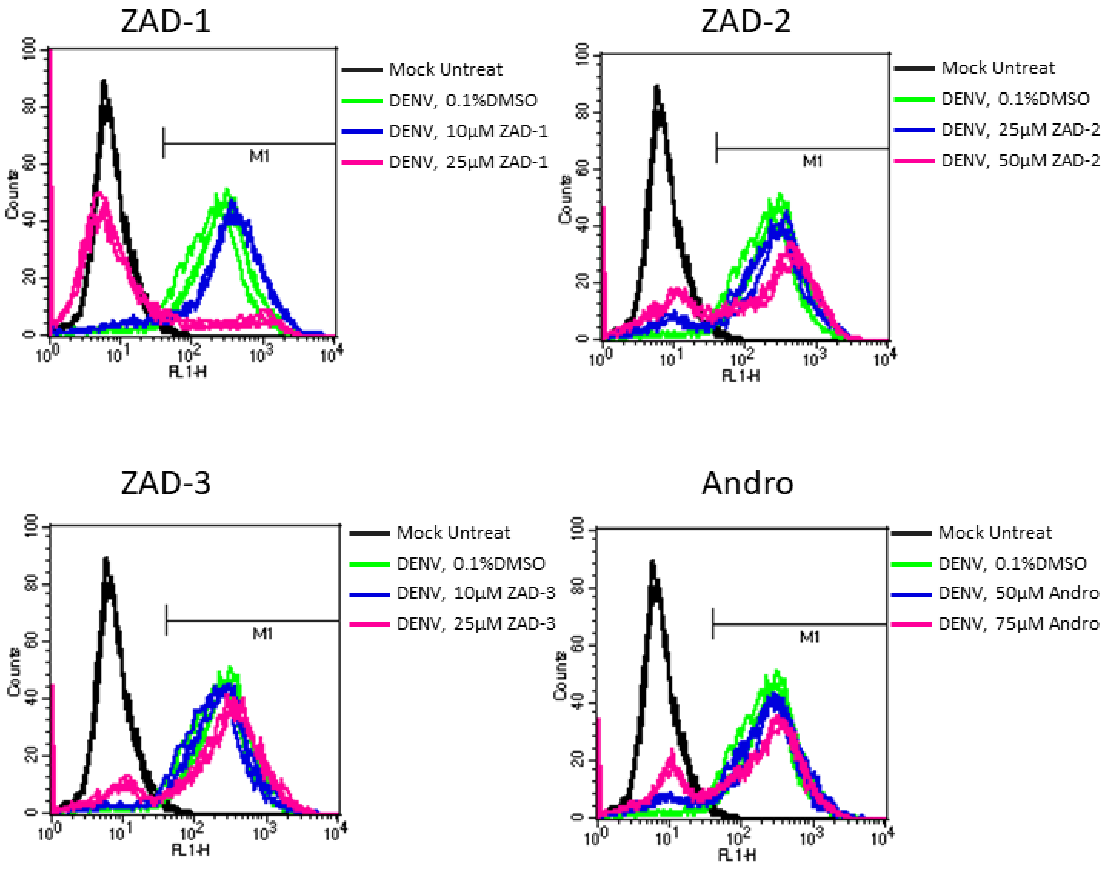

Histogram of figure 3(C)

Figure S15. Raw flow cytometry data from Figure 3C.

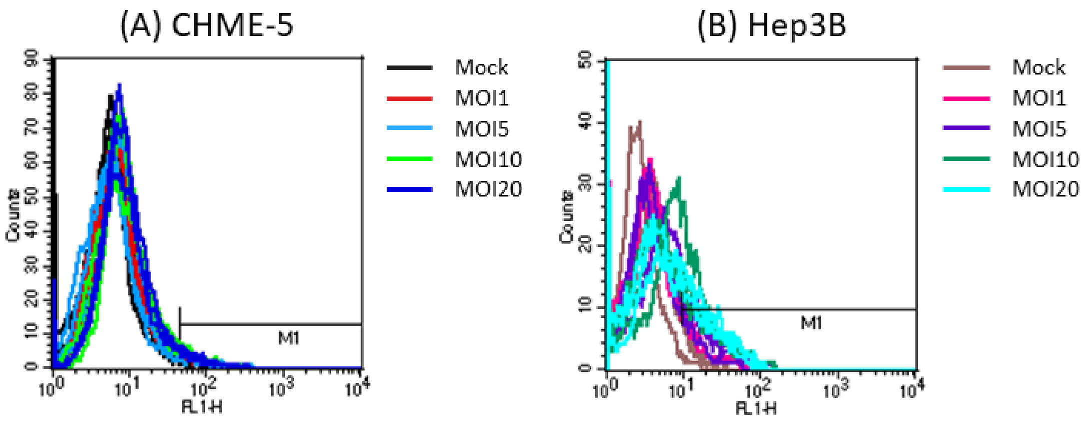

Histogram of figure S1(A) and S1(B)

Figure S16. Raw flow cytometry data from Figure S1A and S1B.

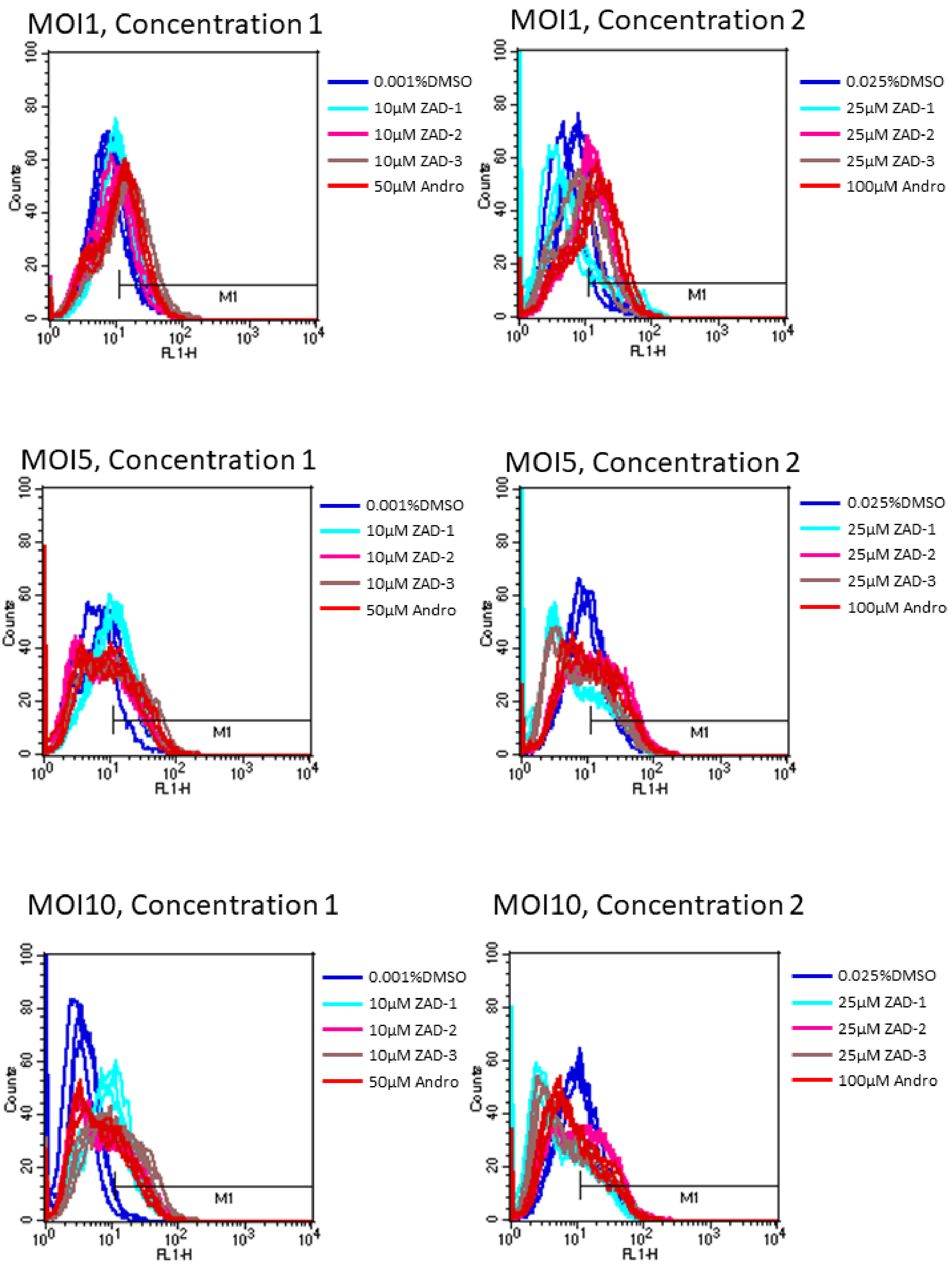

Histogram of figure S8

Figure S17. Raw flow cytometry data from Figure S8.

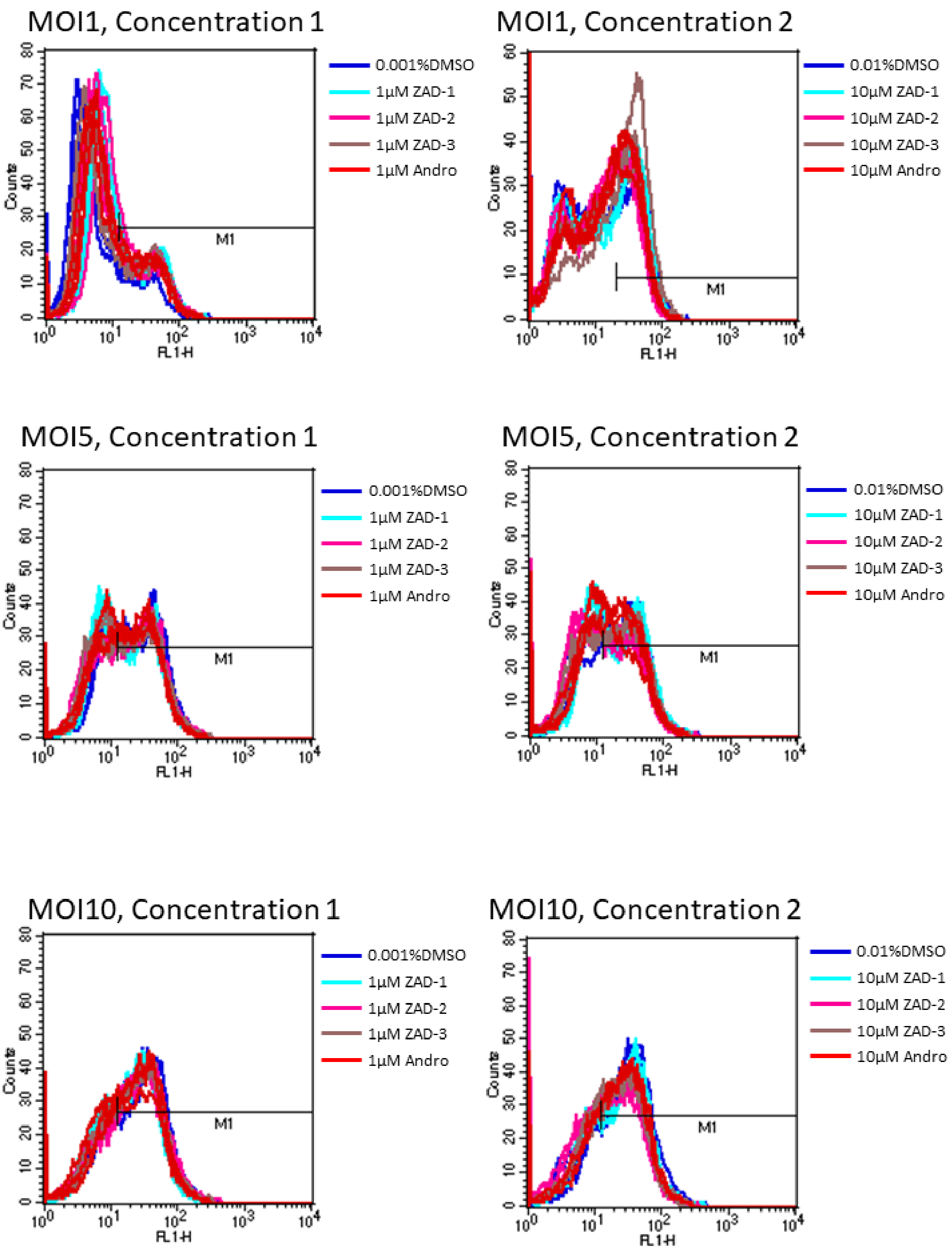

Histogram of figure S9

Figure S18. Raw flow cytometry data from Figure S9.

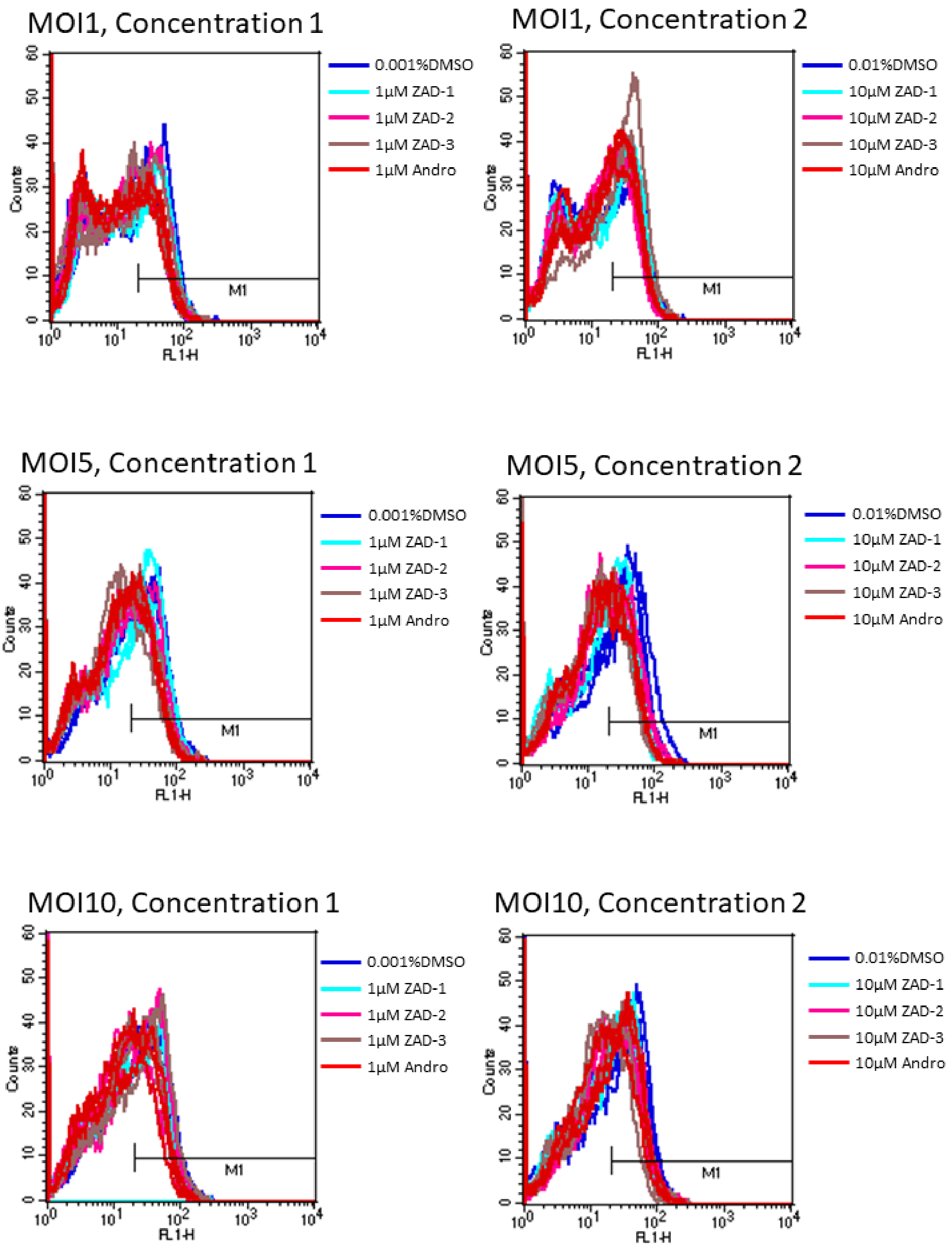

Histogram of figure S10

Figure S19. Raw flow cytometry data from Figure S10.

General information for chemistry

^{1}H and ^{13}C NMR spectra were recorded on a Bruker AV-400 spectrometer at 400 and 100 MHz, respectively, in $CDCl_3$, CD_3OD, $(CD_3)_2SO$ and C_6D_6 as indicated. Coupling constants (J) are expressed in hertz (Hz). Chemical shifts (δ) of NMR are reported in parts per million (ppm) units relative to the solvent. The high resolution of ESI-MS was recorded on an Applied Biosystems Q-STAR Elite ESI-LC-MS/MS mass spectrometer, respectively. Unless otherwise noted, materials were obtained from commercial suppliers and used without further purification. Melting points were measured using an YRT-3 melting point apparatus (Shanghai, China) and were uncorrected. HPLC conditions for analysis of the purity of ZAD-1, ZAD-2 and ZAD-3: Sunfire C18 column (4.6 × 250 mm, 5 μm), elution by 80% methanol with 20% purified water, rate = 0.8 ml/min, detection wavelength of 220 nm.

^{1}H NMR of **ZAD-1**

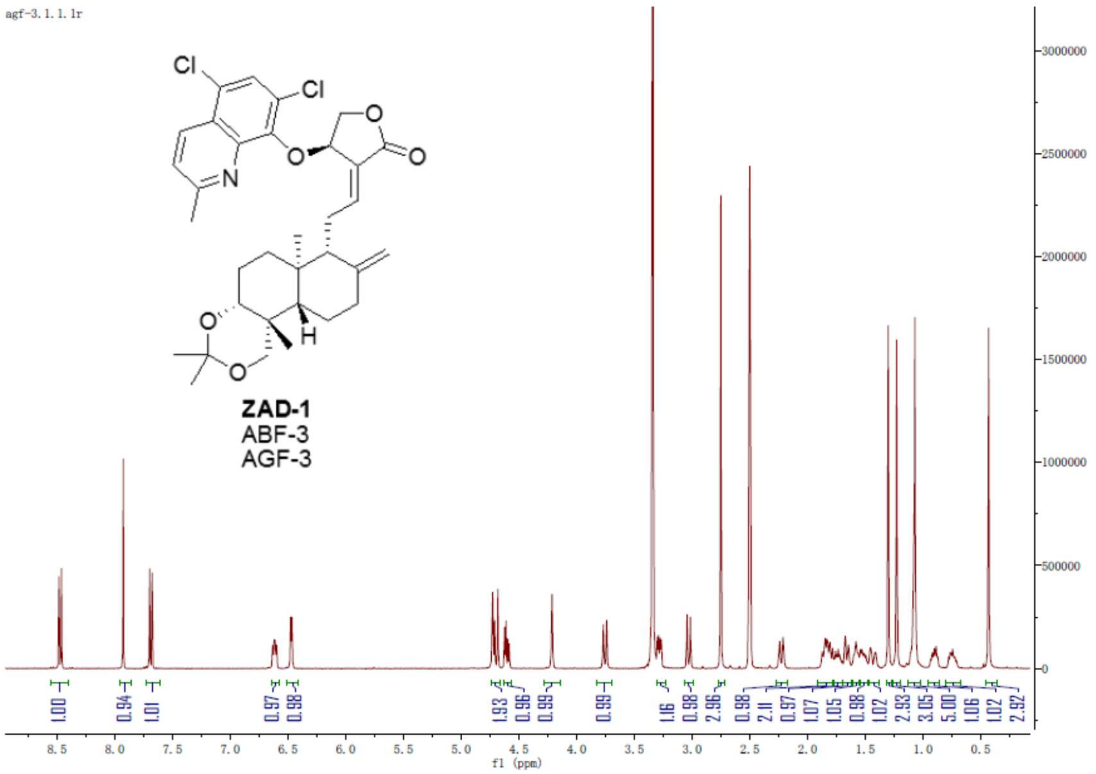

^{13}C NMR of **ZAD-1**

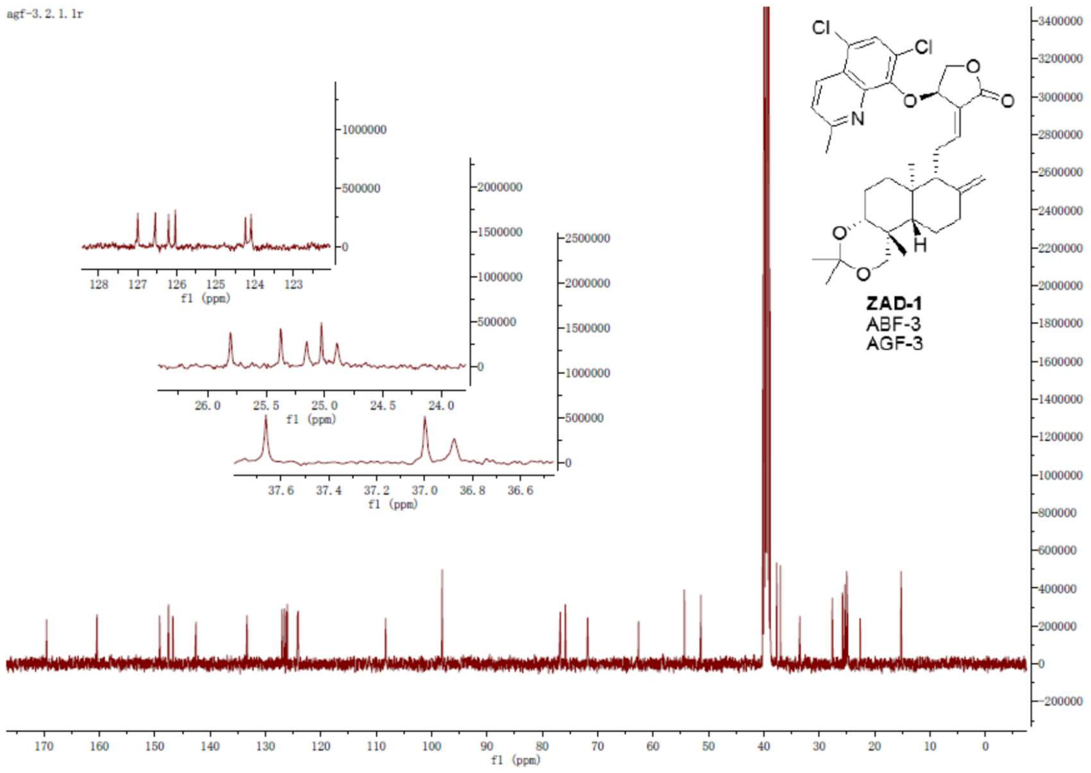

ZAD-1 purity (>98%) by HPLC

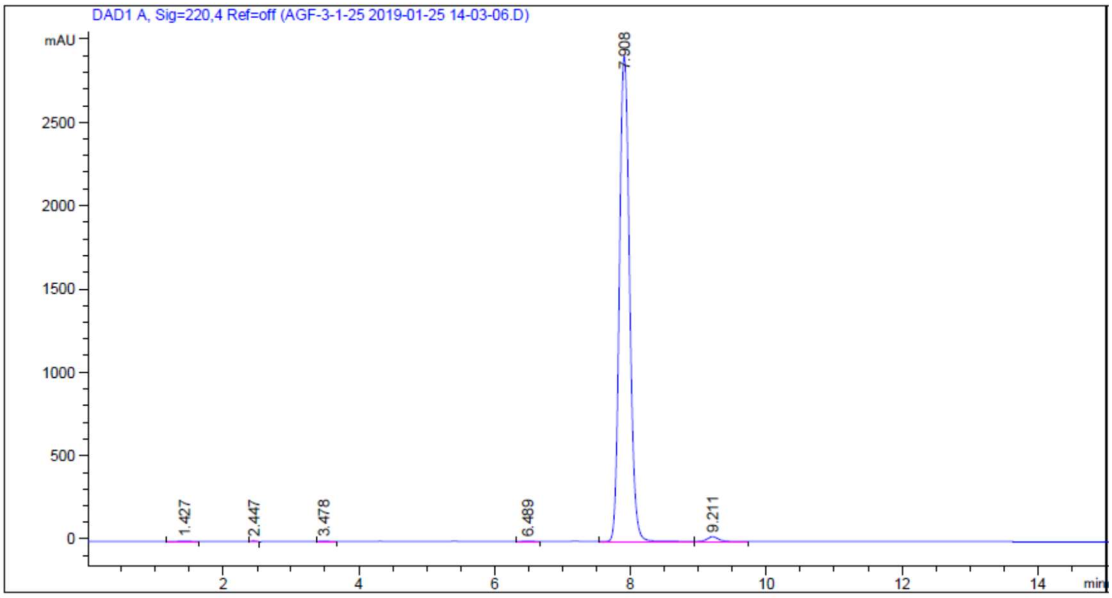

^{1}H NMR of **ZAD-2** (Sci Rep 2017, 7: *4738*)

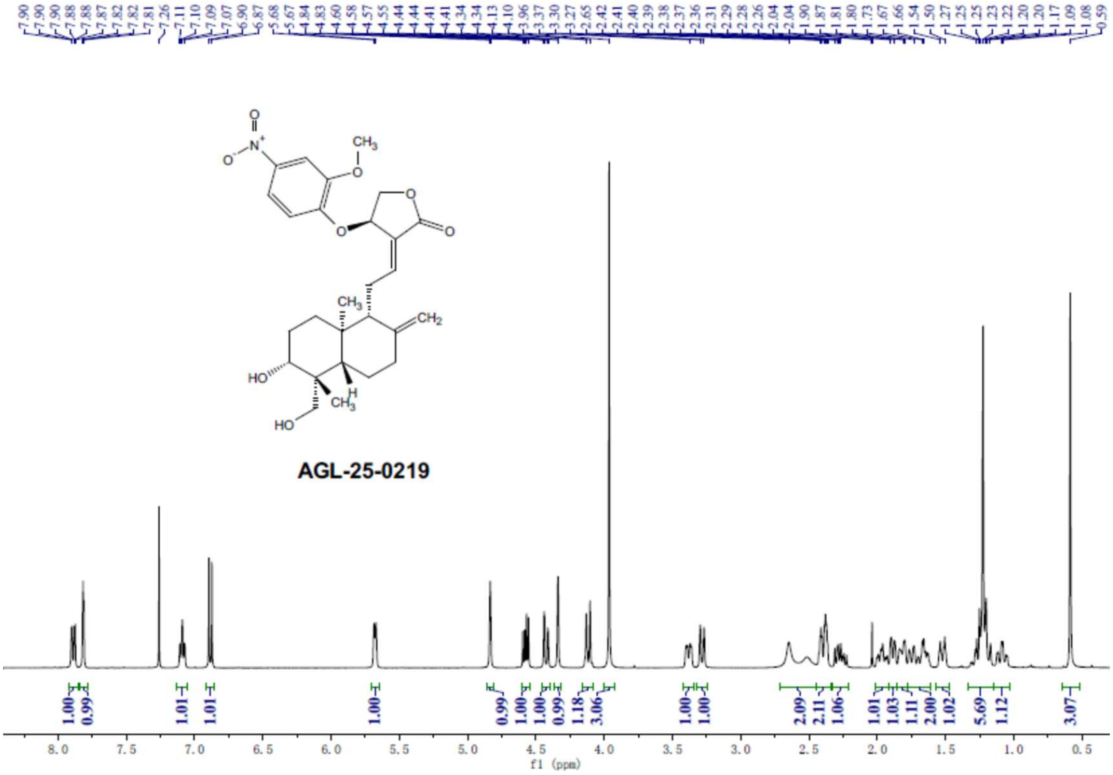

^{13}C NMR of **ZAD-2** (Sci Rep 2017, 7: *4738*)

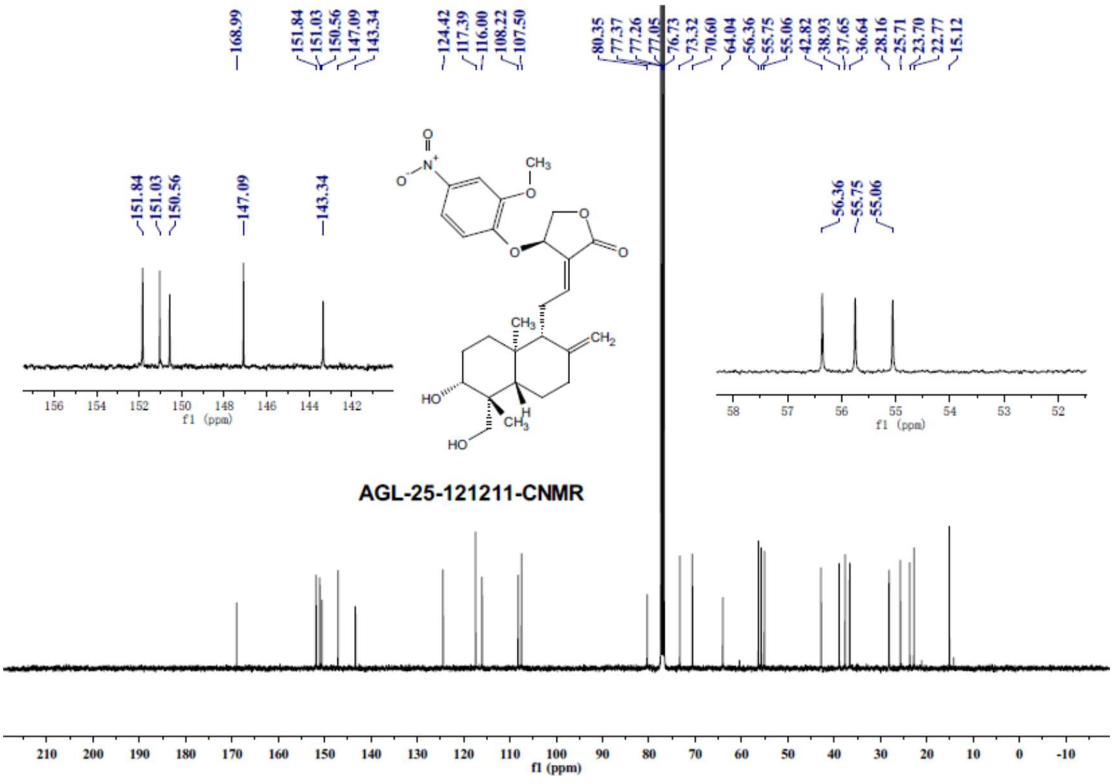

ZAD-2 purity (>98%) by HPLC (Sci Rep 2017, 7: *4738*)

Sunfire C18 column (4.6 × 250 mm, 5 μm), elution by 80% methanol with 20% purified water, rate = 0.8 ml/min, detection wavelength of 220 nm.

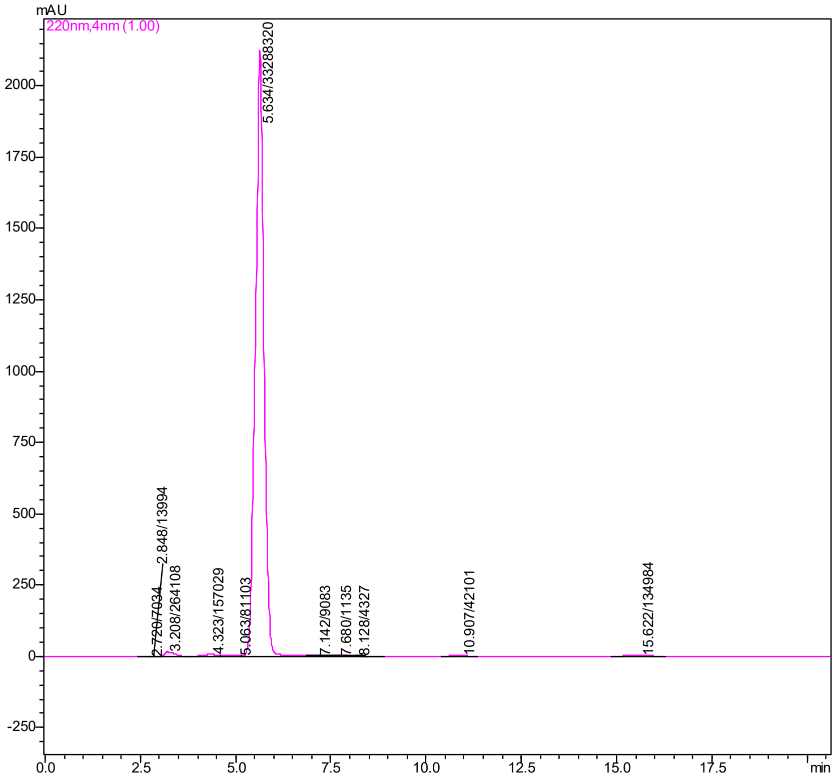

^{1}H NMR of **ZAD-3** (Sci Rep 2017, 7: *4738*)

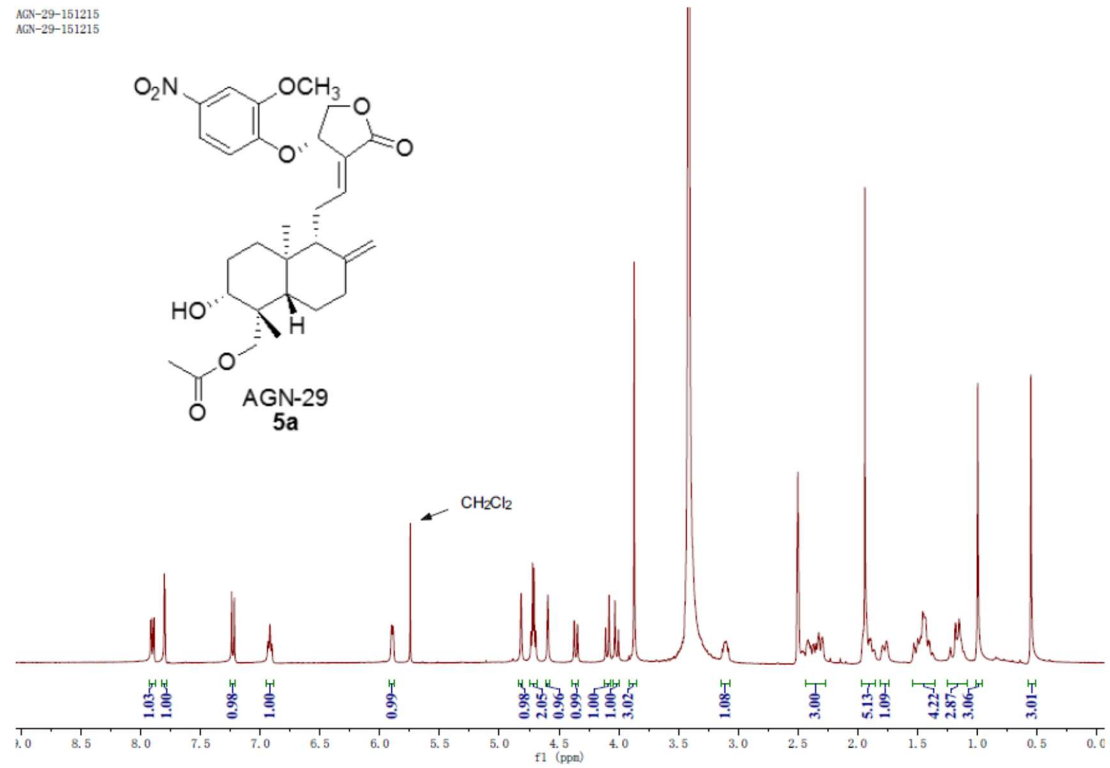

^{13}C NMR of **ZAD-3** (Sci Rep 2017, 7: *4738*)

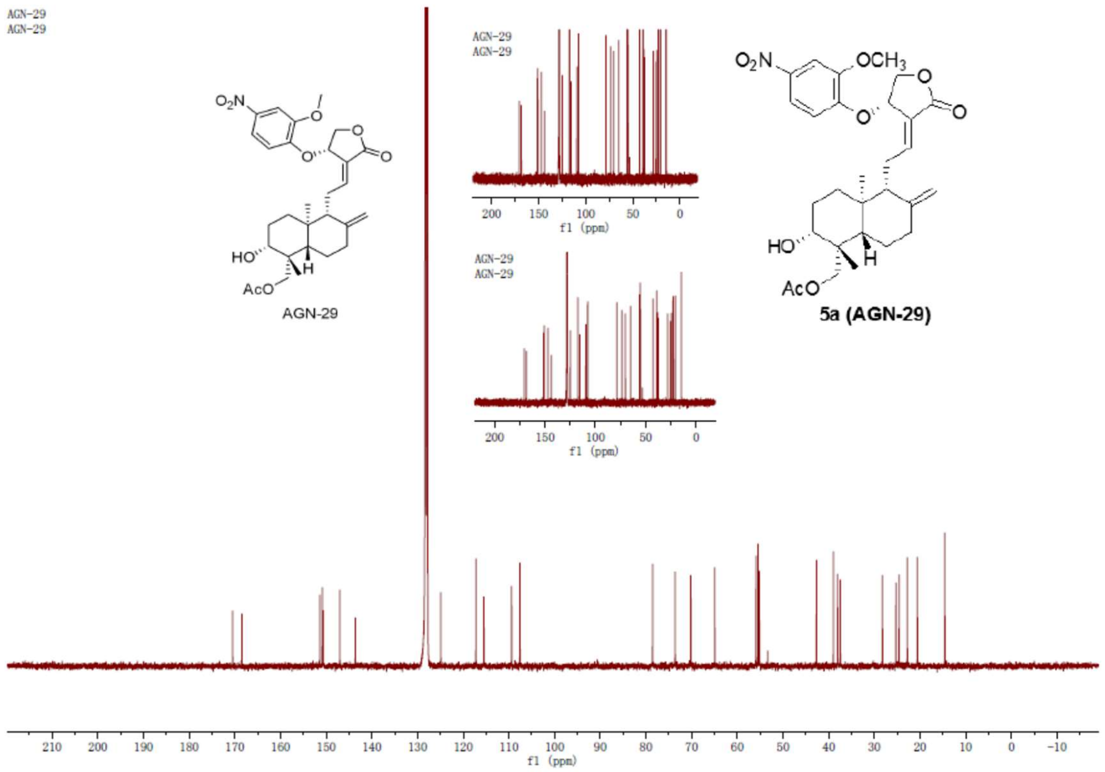

ZAD-3 purity (>98%) by HPLC (Sci Rep 2017, 7: *4738*)

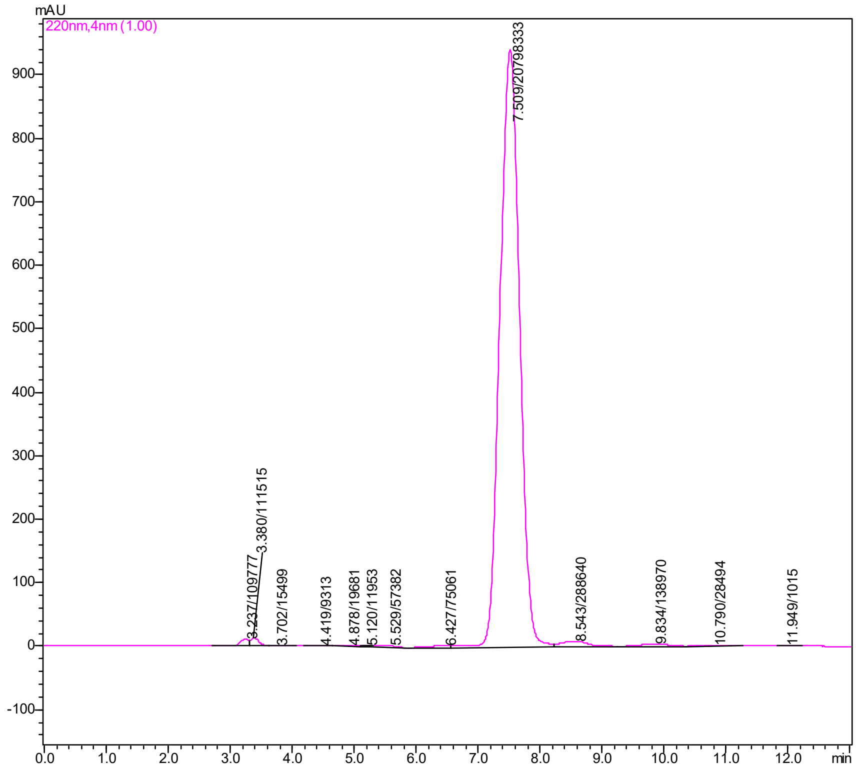

Supplement: Supplementary file 1 [file molecules-25-05037-s001.pdf]
